# Implementation of a batched stepped wedge trial evaluating a quality improvement intervention for surgical teams to reduce anastomotic leak after right colectomy

**DOI:** 10.1186/s13063-023-07318-9

**Published:** 2023-05-15

**Authors:** Mary L. Venn, Charles H. Knowles, Elizabeth Li, James Glasbey, Dion G. Morton, Richard Hooper, ESCP EAGLE Safe Anastomosis Collaborative

**Affiliations:** 1 Barts and the London School of Medicine and Dentistry, Queen Mary University of London, Blizard Institute, 4 Newark Street, London, E1 2AT UK; 2 University of Birmingham, Rm 31, Fourth floor, Heritage Building, Academic Department of Surgery, Birmingham, B15 2TT UK; 3 NIHR Global Health Research Unit on Global Surgery, Institute of Translational Medicine, Heritage Building, Birmingham, B15 2TH UK; 4 Barts and the London School of Medicine and Dentistry, Queen Mary University of London, Institute of Population Health Sciences, 58 Turner Street, London, E1 2AB UK

**Keywords:** Stepped wedge trial, Batched stepped wedge, Incomplete trial design, Cluster randomised trial, Group randomised trial, Colorectal surgery, Anastomotic leak prevention

## Abstract

**Background:**

Large-scale quality improvement interventions demand robust trial designs with flexibility for delivery in different contexts, particularly during a pandemic. We describe innovative features of a batched stepped wedge trial, ESCP sAfe Anastomosis proGramme in CoLorectal SurgEry (EAGLE), intended to reduce anastomotic leak following right colectomy, and reflect on lessons learned about the implementation of quality improvement programmes on an international scale.

**Methods:**

Surgical units were recruited and randomised in batches to receive a hospital-level education intervention designed to reduce anastomotic leak, either before, during, or following data collection. All consecutive patients undergoing right colectomy were included. Online learning, patient risk stratification and an in-theatre checklist constituted the intervention. The study was powered to detect an absolute risk reduction of anastomotic leak from 8.1 to 5.6%. Statistical efficiency was optimised using an incomplete stepped wedge trial design and study batches analysed separately then meta-analysed to calculate the intervention effect. An established collaborative group helped nurture strong working relationships between units/countries and a prospectively designed process evaluation will enable evaluation of both the intervention and its implementation.

**Results:**

The batched trial design allowed sequential entry of clusters, targeted research training and proved to be robust to pandemic interruptions. Staggered start times in the incomplete stepped wedge design with long lead-in times can reduce motivation and engagement and require careful administration.

**Conclusion:**

EAGLE’s robust but flexible study design allowed completion of the study across globally distributed geographical locations in spite of the pandemic. The primary outcome analysed in conjunction with the process evaluation will ensure a rich understanding of the intervention and the effects of the study design.

**Trial registration:**

National Institute of Health Research Clinical Research Network portfolio IRAS ID: 272,250. Health Research Authority approval 18 October 2019. ClinicalTrials.gov, identifier NCT04270721, protocol ID RG_19196.

## Background

Improvement of surgical safety is a global healthcare priority with significant potential to reduce morbidity and mortality. The most important complication of colorectal surgery is anastomotic leak [1–3]. This can occur when a joint made between two ends of the bowel, e.g. following the removal of part of the bowel, fails to seal properly resulting in leakage of bowel contents into the peritoneal cavity, infection, sepsis or even death. The ESCP sAfe Anastomosis proGramme in CoLorectal SurgEry (EAGLE) study is an international, pragmatic, cluster randomised trial of a quality improvement intervention designed to reduce anastomotic leak following a common operation called right colectomy (removal of the right side of the large bowel, to treat bowel cancer or benign disease, and connect small bowel to remaining colon), by implementation of an education programme [4].

Previous research has shown that specific risk factors that may be identified pre- and intra-operatively increase the risk of anastomotic leak after right colectomy, and variation in surgical practice can harm patient outcomes [1, 5]. The EAGLE intervention promotes pre- and intra-operative operative risk calculation and harmonisation of surgical practice.

Large-scale quality improvement interventions generate a multitude of challenges that demand a robust study design and the ability for the design to adapt for delivery in different environments and social contexts. The EAGLE trial adopted an innovative batched stepped wedge design aimed at achieving good uptake and statistical efficiency. In this paper, we expand on the features of this design and reflect on lessons learned about the implementation of randomised evaluations of quality improvement programmes on an international scale.

## Methods

### Summary of the EAGLE trial

**Fig. 1:**
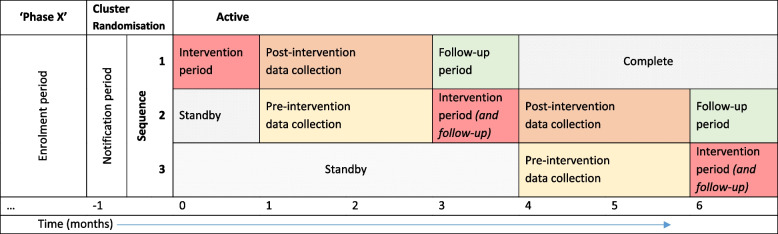
Incomplete stepped wedge study design timeline. Example timeline for a single phase or batch in the trial. The figure shows the incomplete dog-leg study design. Sequence randomisation takes place when clusters have enrolled and are ready to begin the study. This model is repeated multiple times with ‘time 0’ being reset for each new batch. Note: new patients are collected for 2 months in each data collection period with outcomes followed up to 30 days after

Any surgical units that routinely perform both elective and emergency right colectomy in adult patients were eligible to enrol on the study. A surgical consultant, surgical trainee, an anaesthetist and a nurse principle investigator were required as a minimum in each local team (cluster). The primary objective was to reduce 30-day anastomotic leak rate following right colectomy. Clusters were randomised in a series of batches or phases approximately every 2 months, provided at least 18 clusters were ready (batches varied in size). In each batch, clusters were randomised 1:1:1 to three different sequences, with data collection in two distinct 2-month calendar periods (Fig. 1). The sequences differed in terms of the timing of implementation of the intervention, and in the periods when data were collected. In the terminology of cluster randomised trial design, each batch consisted of an incomplete, three-sequence stepped wedge design [6], and the trial as a whole was an example of a batched (incomplete) stepped wedge design [7]. We expand further on these concepts below. The plan for analysis, briefly, was to analyse each batch separately and pool (meta-analyse) the estimates of intervention effect (further details of the analysis plan are provided in the published protocol) [4].

The hospital-level intervention required all surgeons to complete online education modules and then with their surgical teams to implement the ‘ESCP Safe Anastomosis Intervention’. The intervention was comprised of three components: pre-operative risk stratification, harmonisation of surgical technique and an intra-operative ‘ESCP Safe Anastomosis checklist’. Data collection involved identification and enrolment of all consecutive patients ≥ 18 years undergoing right colectomy with or without anastomosis in the defined data collection period, and follow-up until 30 postoperative days. As this was a hospital-level educational intervention, patient-level consent was not necessary in most jurisdictions. EAGLE was prospectively registered, IRAS ID:272,250; ClinicalTrials.gov, identifier NCT04270721 and the full protocol is published elsewhere [4].

### Choosing elements for quality improvement

EAGLE was tailored specifically to tackle differences identified in patient outcomes and improve overall anastomotic leak rate. Previous research including European Society of Coloproctology (ESCP) prospective audits demonstrated patient characteristics and intra-operative factors that increased the likelihood of anastomotic leak as well as differences in patient outcomes depending on whether they were operated by a general or colorectal surgeon [1, 5, 8]. Directly addressing the lessons learned in existing studies, the EAGLE Protocol Working Group created the EAGLE programme to ‘level up’ care by quality improvement. The EAGLE intervention adopts first, an objective patient risk calculator [9] to identify patients at increased risk and enable surgeons to reconsider anastomosis and/or a defunctioning ileostomy (to divert bowel contents away from the join and into a bag on the abdominal wall, to prevent internal contamination while an anastomosis is healing). Second, the training modules ensure the best evidence is presented to all surgeons; general or colorectal, to harmonise operative practice and reduce differences in technique. Third, the intra-operative checklist enables factors, identified by the anaesthetist, theatre staff or surgeons, that arise during the operation to be incorporated in final decision-making for the anastomosis.

### Selecting outcomes

The EAGLE study deliberately selected routinely collected data that would be simple to locate for any colorectal patient in any hospital regardless of geography or socio-economic situation. This promotes a high level of data completeness and reduces missing data. Full details of data collection and the case report form can be viewed via the published protocol [4].

### Collaborative networks

The ESCP’s programme of snapshot audits (multicentre prospective audits to generate large datasets about colorectal surgery and its outcomes) [1, 10, 11], created an established international family of surgical researchers familiar with obtaining local governance approvals, collecting and uploading data, and working remotely with a central team. This network helped promote and extend the study as the group had already delivered results and publications from previous work and retained the collaboration of many reputable establishments. In addition, the ESCP network allowed EAGLE to access a patient population, in which rates of anastomotic leak are established and would be comparable; notably, the 2015 snapshot audit reported an 8.1% anastomotic leak rate following right colectomy in > 3000 patients [1].

### Batched stepped wedge design

Large clinical trials are notoriously complex to deliver and can have prolonged timelines. Surgical researchers are increasingly interested in methodological developments in randomised trials [12]. EAGLE planned to recruit 333 clusters (surgical units) and needed an approach that would allow clusters to on-board the trial as soon as they were ready, to avoid delays during which enthusiasm could start to wane. Stepped wedge trials typically involve all clusters commencing at the same time, though the possibility of separating clusters in a stepped wedge trial into distinct batches has recently been proposed and formalised to mitigate the problem of delays [7]. Each batch in a batched stepped wedge design can commence at different times, and effects of calendar time are adjusted separately within each batch, but with all batches contributing to the estimation of the intervention effect. In practical terms this allows investigators to focus recruitment of clusters in any given batch, taking optimal advantage of clusters’ availability to participate, while still accruing the overall statistical power needed to answer the research question.

The EAGLE protocol suggested indicatively that 7 batches of around 48 clusters each might be run, but the design and approach to analysis (meta-analysis across batches) gave us the flexibility to have batches of varying sizes. The make-up of a batch was determined largely by the order in which clusters enrolled, but again the design and meta-analytic approach gave us the flexibility, if we wanted it, to focus the recruitment of clusters to different batches in different global regions, for example. The sample size target (see below) was based on the total number of clusters and total number of surgical procedures across all batches.

### Statistical efficiency

An incomplete stepped wedge design was chosen in order to improve statistical efficiency by reducing both the number of clusters and the burden of individual-level data collection over all clusters necessary to achieve the required power [13]. The particular incomplete design adopted for each batch of the EAGLE trial was a “dog-leg” design with three randomised sequences and two periods of data collection [13] as demonstrated in Fig. 1. In the first sequence the intervention is implemented before any data collection takes place, and data are collected in the first period only. In the second sequence data are collected in both periods, and the intervention is implemented between these two periods. In the third sequence the intervention is not implemented until after data collection, and data are collected in the second period only. All clusters receive the intervention within 28 weeks.

The EAGLE trial was designed to detect what was judged to be a clinically important, absolute reduction in anastomotic leak rate from 8.1 to 5.6% (relative risk reduction 30%), based on the leak rate in previous research as above [1]. In our sample size calculation we assumed an intra-cluster correlation coefficient of 0.05 and a mean identification rate of 10 procedures per 8-week data collection period, and on this basis calculated an overall “design effect” of 0.9 for our cluster-randomised dog-leg design relative to an individually-randomised, single-period, parallel-groups design [14]. This design effect is an inflation factor (or in this case a deflation factor) that can be applied to the number of clusters required to achieve the specified power [12]. The design effect is less than 1.0 in this case because of the efficiency offered by having both pre- and post-intervention data collection from the same clusters in the second sequence. Thus we determined that we needed 333 clusters with data on a total of 4440 surgical procedures in order to achieve 80% power at the 5% significance level. This calculation included a correction to allow for variable cluster size (for further details of the sample size calculation, see the published protocol) [4].

We also calculated that if we had used a more conventional, parallel groups cluster randomised design, with data collection in both periods in all clusters, we would have needed 358 clusters with data on 7,160 surgical procedures in total, thus demonstrating the superior efficiency of the dog-leg design.

### Flexibility for clusters and participants

The EAGLE protocol and resource packs offered a flexible approach to data collection to respect differences in local practice, with hard copy or electronic case report forms and the option for contemporaneous or retrospective database data entry. Password-protected e-modules were available throughout a four-week intervention period and thereafter to enable participants to access them at their convenience rather than fixed, timed lectures.

### Maximising scholarship

*Every* surgeon consultant or trainee who could be the primary operator for a right colectomy in emergency or planned cases was encouraged to complete the e-modules in *every* collaborating hospital. Access was enabled for the start of the EAGLE intervention period at each cluster. Module completion generated Continuing Professional Development points, accredited by the Royal College of Surgeons of England, free of charge. Even clusters (hospitals) randomised to submit only pre-intervention patient data later received the intervention, such that no clusters completed the study without the opportunity for all surgeons to access the evidence-based resources. Module access continues beyond the timeframe of the study for users to refer back.

### Language

EAGLE’s global invitation introduced some challenges for the Central Operations Committee to ensure relevant resources were accessible to all participants. Theatre team presentations and the ESCP Safe Anastomosis Checklist were translated to thirteen languages (independently and checked by national coordinators) and hosted at a single hub. A working level of English was assumed however for the surgical team, with the e-modules for surgeons only available in English.

### Case ascertainment and data accuracy

Data validation was addressed with two main strategies. Stepped wedge cluster randomised trials demand a particularly rigorous approach to case ascertainment [15]. All participating EAGLE clusters were contacted after batch completion to report the number of sequential eligible patients in a randomly-selected 2-week sample of their data collection periods. Figures were compared with the cases reported electronically and any inconsistencies were investigated with the whole period being re-checked, any missed cases added or erroneous cases removed. Additionally, data accuracy was assessed at three clusters per randomisation sequence (approximately 30% clusters in each batch), selected using a random number generator. Each cluster nominated someone to complete the data accuracy exercise, resubmitting ten key data points for up to the first ten consecutive patients in a single data collection period.

### Trial steering committee and data monitoring committee

EAGLE constituted a Trial Steering Committee (TSC) and Data Monitoring Committee (DMC) to provide ongoing oversight and independent decision-making in response to challenges. The DMC had access to unblinded, interim data summaries from wholly and partially completed batches of the trial, and their remit included monitoring the assumptions of the original sample size calculation. They also had the power to recommend to the TSC that the entire trial be stopped early, or that a single ongoing batch be terminated. The TSC acted in the usual role of “critical friend” [16], had access to blinded, interim summaries of data, and advised on when the trial should be considered to be complete.

## Results

In the following sections, we reflect further on the evolution of the trial processes as the trial unfolded.

### Effects of and resilience to COVID-19

EAGLE recruited and randomised the first batch of hospitals in December 2019, as ‘phase 1’, and the second in February 2020, ‘phase 2’, prior to the start of the COVID-19 pandemic. The pandemic rapidly shut down global surgical services and a decision was made to suspend the EAGLE study, just five weeks after launch, on 13^th^ March 2020. As pandemic waves reached different countries at different times, some clusters continued to progress local approvals and as the first wave retreated, clusters were asked their readiness and capacity to restart the EAGLE programme. ‘Phase 1’ resumed on 27^th^ July 2020 with a mix of hospitals derived from phases 1 and 2 that had indicated readiness. Clusters who could not yet participate joined later batches.

Clusters in ‘phase 1’ that had implemented the intervention but then paused due to COVID-19 were offered a 2-week ‘refresher’ intervention period for teams to review e-modules and intervention components before embarking on post-intervention data collection. Several clusters reported structural or organisational changes related to the pandemic and EAGLE adapted to ensure inclusivity despite these circumstances. One hospital that had previously performed both elective and emergency colorectal surgery was re-structured with its sister hospital to separate emergency operating at one site and elective operating at the other, but with a single, cross-site surgical team. On resumption of the study, local approvals were amended to include both sites as a single cluster. Conversely, in another site where there were two entirely separate surgical teams, each team was randomised as a separate cluster.

Although the interruption to the first batches impacted on the conduct of the trial and thus potentially the interpretation of the data in these batches, this impact was contained and did not compromise later batches, illustrating a more general robustness in the batched approach to design. When the trial data are analysed it may be necessary to perform a sensitivity analysis excluding phases 1 and 2.

### Team structure

**Fig. 2:**
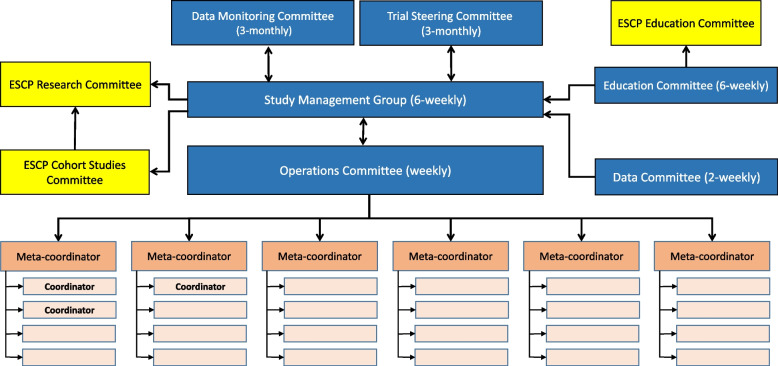
EAGLE team structure showing relationships between committees

The EAGLE team comprised separate Protocol Writing and Education Committee groups to drive forward the study design and the educational resources independently. The Operations Committee was convened to deliver the study with ‘Meta-coordinators’ liaising between the central team and ‘Coordinators’ who were responsible for day-to-day liaison with collaborators from individual clusters, loosely divided into geographical groups of hospitals or by language (Fig. 2).

EAGLE Coordinators worked with local Principal Investigators (PIs) to ensure that relevant local approvals were realised. Any regional or national ethical approvals were shared with other members of the same jurisdiction to minimise duplication of work. Resources and approvals were disseminated by close communication between PIs, coordinators and meta-coordinators.

## Discussion

### Trial strengths

EAGLE has proved a highly effective study design to deliver a complex quality improvement intervention in a wide range of settings across multiple study batches. Translation of resources and batching of clusters has enabled the study to navigate geographic, language and varied pandemic landscapes. The streamlined outcomes and design with incomplete cross sections offered high data completeness with low investigator burden. The trial was powered to efficiently detect reduction of a significant, measurable outcome. Finally, regardless of whether the primary outcome is achieved, the trial delivered a framework for education and operating team communication to hospital teams in all study sequences.

### Limitations

Implementing the design in batches had a number of practical benefits [7, 17], including enabling sequential participation of different clusters through the course of the trial. The use of an incomplete rather than a complete design in each batch had a theoretical advantage in terms of the quantity of data required for the trial, but there were also disadvantages.

The staggered start dates for data collection and introduction of the intervention meant that collaborators, e.g. Surgical Trainee Principal Investigators could miss participating in the study itself despite garnering the appropriate approvals, because the lead-in time after randomisation could be up to 20 weeks. This affected trainees or other collaborators moving between hospitals/departments, particularly in sequence 3. The risks with such a long lead in time include reduced motivation and engagement by the time study activities begin; however, this may also be seen in the context of studies that recruit many clusters to start at the same time, as those recruited first may have lost motivation by the time enough teams are prepared to start. Where long delays mean collaborators have changed departments or hospitals, the local team also needs to recruit new core team members who may not be as familiar with the study.

A further risk associated with long lead-in times before pre-intervention data collection is the risk that participants will, in the meantime, access educational materials from elsewhere. This will, at worst, attenuate our estimate of the effect of the intervention.

EAGLE sequence 3 collaborators must also rely on internal rather than external motivation to complete e-learning, (since there was no post-intervention data collection for this group therefore core EAGLE team members would have less reason to urge surgeon colleagues to complete the modules). While this would not affect the study’s primary objective (as no post-intervention data collection planned), it would reduce the dissemination of evidence-based education content which is designed to be delivered at every EAGLE participating cluster.

The e-modules contained extensive evidence-based content for surgeons that were presented in English. Most surgeons in the ESCP are accustomed to working in English however the resource was offered to all surgeons at participating hospitals and this monolingual resource may have excluded selected surgeon users. In some countries (South Korea, Romania), surgeons formed working groups to undertake the e-modules together. A lead surgeon could translate and interpret the modules, and cross the language barrier for colleagues with more limited English.

One further drawback is that this study did not examine the longer-term effects of the intervention. Individual surgeons or surgical teams could slip back into older less evidence-based practice, team members will inevitably change, and without deliberate maintenance of the practice change, any beneficial intervention effects may be lost. There is a strong case for a follow-up cohort study to assess longer-term adherence and patient outcomes, should the study show a patient benefit.

### Process evaluation

To ensure EAGLE’s results can be understood, a prospectively designed process evaluation is being undertaken. The parallel process evaluation will demonstrate how the education resources have reached learners (engagement), to what extent patients have received, or been exposed to, the EAGLE Safe Anastomosis Programme in the post-intervention groups (implementation), and help identify factors that have promoted or distracted intervention implementation. This will allow the project’s outcomes to be examined, to delineate motivators and barriers for the intervention and to decipher any intervention failure from an implementation failure. This analysis can enhance a study’s overall results and avoid drawing false conclusions of failure (or success) in either aspect of study delivery.

## Conclusion

EAGLE’s robust but flexible trial design allowed sequential recruitment and completion of the study across geographical locations in spite of the pandemic. EAGLE’s collaborative group originating in the ESCP network, helped nurture strong working relationships with resource sharing to reduce duplication. The primary outcome analysed in conjunction with the process evaluation will ensure a rich understanding of the intervention and the effects of the study design.

## Data Availability

Data sharing not applicable to this article as no datasets were generated or analysed during the current manuscript.

## Abbreviations

DMC: Data Monitoring Committee
ESCP: European Society of Coloproctology
EAGLE: ESCP sAfe Anastomosis proGramme in CoLorectal SurgEry
PIs: Principal Investigators
TSC: Trial Steering Committee
